# The Interprofessional Clinical Experience: Introduction to Interprofessional Education Through Early Immersion in Health Care Teams

**DOI:** 10.15766/mep_2374-8265.10564

**Published:** 2017-03-30

**Authors:** Fatema Haque, Michelle Daniel, Michael Clay, Jennifer Vredeveld, Sally Santen, Joseph B. House

**Affiliations:** 1 Program Manager of the Clinical Trunk, Office of Medical Student Education, University of Michigan Medical School; 2 Assistant Dean for Curriculum, University of Michigan Medical School; 3 Clinical Assistant Professor of Emergency Medicine, University of Michigan Medical School; 4 Assistant Professor of Internal Medicine, University of Michigan Medical School; 5 Clinical Assistant Professor of Internal Medicine and Pediatrics, University of Michigan Medical School; 6 Assistant Dean for Educational Research and Quality Improvement, University of Michigan Medical School; 7 Professor of Emergency Medicine and Learning Health Sciences, University of Michigan Medical School; 8 Assistant Professor of Emergency Medicine and Pediatrics and Communicable Diseases, University of Michigan Medical School; 9 Director of Interprofessional Education, University of Michigan Medical School

**Keywords:** Reflection, Interprofessional Education, Early Clinical Experience, Interprofessional Practice, Initial Clinical Experience, Health Care Teams

## Abstract

**Introduction:**

The ability to collaborate as a member of interprofessional teams is essential for patient care and a core competency for students in health professions education. We developed a yearlong course, the Interprofessional Clinical Experience (ICE), to introduce first-year medical students to team-based aspects of the health care environment and provide them with a foundation upon which later experiences can grow.

**Methods:**

The course uses experiential learning and critical reflection through reflective writing to orient students to working with care teams. Students receive assessments from faculty and various health care professionals. The course requires students to describe the roles and responsibilities of a variety of health care professionals, utilize effective communication with other health professionals on health care teams, demonstrate the ability to work on an interprofessional team, and examine their own and others’ perspectives by engaging in self-directed learning and reflective practice.

**Results:**

Annual course evaluations revealed that the majority of students agreed or strongly agreed that ICE contributed to their understanding of the health care team's roles and improved their ability to communicate with health care professionals, their understanding of health care systems, and their ability to work on an interprofessional team. The course also provides curricular content for the newly implemented Liaison Committee for Medical Education's accreditation requirement on interprofessional collaborative skills.

**Discussion:**

The first implementation of this resource demonstrated that students met the educational objectives of the ICE and gained a better sense of the health care system and teams.

## Educational Objectives

By the end of the course, students will be able to:

1. Describe the roles and responsibilities of a variety of health care professionals.
2. Utilize effective communication with other health professionals on health care teams.
3. Develop an understanding of the basic organization of health care systems.
4. Examine their own and others’ perspectives by engaging in self-directed learning and reflective practice.

## Introduction

The ability to effectively collaborate as a member of interprofessional teams is essential for patient care and a core competency for students in health professions education.^1,4^ Effective collaboration includes “demonstrating respect for other professions, understanding their roles, communicating clearly and effectively, resolving conflict effectively, and sharing common goals.”^3^ Interprofessional education, defined as curricular experiences where two or more professions learn with, from, and about each other, can facilitate effective collaboration.^4^

The Interprofessional Clinical Experience (ICE) is a yearlong course designed to introduce preclinical students to team-based aspects of the health care environment through interprofessional education and provide them with a foundation upon which later experiences can grow. The course is intended for first-year medical students and would be suitable for any preclinical students in other health science fields, such as nursing, dentistry, pharmacy, kinesiology, and so on. Through a half-day orientation and biweekly longitudinal experiences with a variety of health care professionals in inpatient and outpatient settings, ICE introduces students to interprofessional practice, systems-based medical practices, and varying compositions of health care teams. The longitudinal nature of the course allows students to establish formative relationships with personnel in the clinical environment, develop a larger view of both inpatient and outpatient clinics, and think about the broader health care system.

ICE also provides documentation for the newly implemented Liaison Committee for Medical Education's accreditation standard, interprofessional collaborative skills, which requires medical schools to prepare their students to function collaboratively on health care teams.^5^ Through direct interaction with practicing health professionals from other disciplines, students gain insight into health care team interactions and how these teams provide coordinated services to patients.

Finally, ICE takes a different approach to interprofessional education. Most interprofessional education curricula are focused on bringing students from various health care disciplines together for discussion or simulation exercises for a single day or a small series of events.^6,4^ In contrast, ICE provides learners with meaningful, longitudinal, interprofessional experiences with practicing health care providers in clinical settings.

## Methods

The target audience for this resource is first-year medical students or preclinical students in health science fields such as nursing, dentistry, kinesiology, pharmacy, and so on. There are no prerequisites.

Instructors should start with the ICE instructor packet (Appendix A), which describes in detail the materials for the course and how to use them.

Students should take the prequiz (Appendix B) to help course leadership gain insight into students’ baseline knowledge of various health care professionals and to prime students to areas of interprofessional practice they may be unaware of. This 18-question multiple-choice quiz assesses students’ knowledge of different professionals in the health system. Very likely, it will demonstrate that students have varying degrees of knowledge and awareness of the different professions. When we administered the quiz, scores ranged from 5% to 100%. The quiz was developed by asking allied health professionals, such as nurses, social workers, respiratory therapists, and so on, what they thought medical students and/or physicians should know about their profession.

The clinical introduction session (Appendix C) provides students with an initial overview of some of the roles they will encounter during their longitudinal experience. In a large-group setting, students view an introductory video (Appendix E) of a clinical case that demonstrates how various members of the health care team were involved in patient care. The purpose of the video is to provide a launching point for health professionals to discuss how their profession operates in teams (Appendix D). The video is followed by small groups of 10 to 12 students, with each group rotating through multiple stations for 15-minute intervals. Each station is led by a different health professional, with the ideal list of health professions being child life specialist, clinical social worker, dietitian, medical assistant, nurse, paramedic, pharmacist, physician's assistant, and radiologist. These professionals provide students with key background about their profession and answers questions for a deeper understanding of their roles and responsibilities. An active learning experience, this introductory session allows students to utilize prior knowledge, ask questions, and develop new understanding.

The longitudinal experience requires students to spend 7 half-days in an inpatient setting and 7 half-days in an outpatient setting over the course of 1 academic year. The goal of using both inpatient and outpatient settings is to expose students to differences in care delivery, pace, role of health care professionals, and how health care teams operate in different contexts. Students are instructed to actively observe and interact with someone from another profession with the intent to learn about the professional's roles and responsibilities, style of communication, and how the professional fits into the larger system of health care. After each experience, students require that the person they observed assess them on competencies related to professionalism, communication skills, and teamwork (Appendix M).

The ICE reading list (Appendix F) provides students with context and background information on various health professions that they will encounter during their visits. These readings elaborate on roles, responsibilities, interprofessional teamwork, communication, and other related issues. Students are encouraged to read about the different health professions prior to each session to improve their overall learning experience and assist them in formulating questions for the providers and being more actively engaged during their visit.

Students gather in small groups of 10 to 12 twice per semester to debrief their experiences. Debriefing with others can help students assimilate activities in their cognition and produce long-lasting learning.^8^ These 1-hour sessions are led by faculty small-group facilitators. Prior to the debriefing session, students receive guiding questions (Appendix J) that trigger critical reflection around important themes such as medical errors, decision making, and challenges to interprofessional practice. Critical reflection is defined as a combination of reflection, the “process where we look at an experience, frame it, and derive meaning from it,” and critical thinking as “the ability to evaluate relevant information and opinions gathered in the reflection stage in a systematic, purposeful, efficient manner developing problem solving skills.”^9^ Students also describe and analyze their experiences, find commonalities and differences, and address any questions or concerns that may have come up during the experience.

Critical reflection is documented through reflective writing exercises. Students examine each ICE experience, distill learning, and synthesize their clinical experience with what they learn in other areas of the medical school curriculum. To facilitate reflection and maximize learning, students receive prompts (Appendix G) and a list of guiding questions (Appendix I) organized in the various stages of ICE (preparation, observation, interaction, and articulation/reflection).^10^ The students are encouraged to consider these questions as they engage in ICE activities. Students are also instructed to use the What? So What? Now What? model of reflection (Appendix H). This memorable model asks them to describe an event or some observations, analyze or evaluate that description, and synthesize the information.^11^ It also helps students avoid common pitfalls such as reinforcing stereotypes about differences, developing simplistic solutions to complex problems, generalizing inaccurately based on limited data, and overlooking the most important or significant learning from the experience.^12^ Reflecting after their clinical experience helps students address prior knowledge and assumptions and articulate any practical learning (e.g., clinical or communication skills) or emotional learning (e.g., sense of professional identity).^13^

Students also write two 2- to 3-page end-of-term papers (Appendices K & L) that encourage deep introspection and synthesis of their experiences with their values. They are instructed to consider their experiences as a whole and write about how their awareness of self and others as medical professionals changed over the course of the term; what trends in communication, teamwork, or systems they observed; and what they hope to learn moving forward. They are also asked to explore how their understanding of themselves as professionals has evolved over time and what impact the experience had on their sense of professional identity.

ICE faculty provide written, formative feedback on each of the writing assignments in order to help students recognize reflective moments, make sense of experiences, tolerate uncertainty, and gain insight.^14^

Students can assess the clinical introduction session and the longitudinal component of ICE by completing the course evaluation (Appendix N). This evaluation contains Likert-style questions that address the learning objectives of individual sessions; it also asks students to comment on the overall course experience.

## Results

The goal of this course is to introduce preclinical students to different models of systems-based medical practices, various compositions of the health care team, and individual responsibilities. During the 2015–2016 academic year, our medical school placed 167 students at 18 different inpatient and outpatient clinical sites throughout the health care system. Students worked with professionals representing over 18 different health professions. Course evaluations (Appendix N) were completed by 81 out of the 167 students (48% response rate) and revealed that the majority of students agreed or strongly agreed ICE contributed to their understanding of the health care team and its members’ roles and improved their ability to communicate with health care professionals, their understanding of health care systems, and their ability to work on an interprofessional team (Table 1).

**Table 1.** 2015–2016 Course Evaluation Results

Question	*N* (%)		
	Strongly Disagree/ Disagree	Neutral	Strongly Agree/ Agree
ICE contributed to my understanding of health care team professions and roles.	5 (6%)	6 (8%)	69 (86%)
ICE improved my ability to communicate with health care professionals.	9 (11%)	22 (28%)	49 (61%)
ICE improved my understanding of health care systems.	6 (8%)	7 (9%)	67 (84%)
ICE improved my ability to work on an interprofessional team.	9 (11%)	19 (23%)	53 (65%)

Abbreviation: ICE, Interprofessional Clinical Experience.

Students provided narrative comments about ICE, with many stating that they found working with and learning about other health professions to be a valuable learning experience. One student wrote,

I noticed when I shadowed in the emergency room on my own time that I was paying a lot of attention to the roles of the physician assistants, pharmacists, paramedics, nurses, and social workers. I am 100% sure I would not have done that had I not been in ICE. It was really informative to see how people were involved and during which stages of the patient's stay in the emergency department.

Another student noted,

I really liked the chance to work with so many different healthcare professionals—something I definitely would not have sought out if not for this course. Even though I never observed a physician, I felt like I learned a lot about my own future roles by seeing what other providers were responsible for in the patient care setting.

Comments such as the preceding one demonstrate how ICE is helping students define their identity as a physician.

Narrative comments from students also highlighted some of the limitations of ICE. Because students are placed throughout the health care system, there are some disparities in experiences. For example, students in smaller clinics have fewer professionals to learn from. Some students expressed a desire to be a more engaged member of the health care team instead of primarily shadowing. Finally, students raised concerns over the number of required reflection assignments, stating that “fewer, more meaningful reflections” would be more beneficial.

In a separate evaluation of the clinical introduction session (Appendix N), 96% of students agreed or strongly agreed that the activities increased their understanding of the education and training required for other health professions, and 94% of students agreed or strongly agreed that the activities increased their understanding of the roles of other health professionals in patient care (Table 2). Narrative comments from students supported these numbers: “I am thrilled that University of Michigan Medical School is introducing us to the importance of teamwork and the roles of other healthcare professionals so early,” and “I loved this event and loved hearing directly from all members of the healthcare team.”

**Table 2.** 2015–2016 Clinical Introduction Session Results (*N* = 57)

Question	Strongly Disagree/ Disagree	Neutral	Strongly Agree/ Agree
The clinical introduction session activities increased my understanding of …			
The education and training required for other health professions.	0%	4%	96%
The roles other health professionals play in patient care.	0%	5%	94%

The health care professionals who participated also had positive comments about the session, with the majority expressing that they appreciated the opportunity to participate and found it a worthwhile, rewarding experience.^14^ They commented that students were engaged, asked thoughtful questions, and were interested in learning about their profession. Both students and health professionals provided feedback on ways to improve the session for next year. Students wanted to hear from more health professionals and to acquire a more holistic view of each profession. Health professionals suggested increasing the length of the sessions and changing the video to reflect the roles of more health professions.

Students also requested feedback on competencies such as professionalism, communication skills, and teamwork from the professionals they observed. In the fall term, 1,121 assessment requests were made. Of these requests, 743 were completed, suggesting that on average, an individual student received four to five evaluations.

## Discussion

This resource provides the foundation for an experiential course dedicated to interprofessional education. The first implementation of ICE demonstrated that students were meeting the educational objectives of ICE and gaining a better sense of the health care system and teams. Still, several logistical and pedagogical challenges were encountered in the implementation of ICE.

Logistical challenges included transportation issues for students, choice in clinical site placement, and identification of potential preceptors at clinical sites. We addressed these by providing students with parking passes and coordinating carpooling, allowing some choice in site placement, and cultivating relationships with liaisons at clinical sites in order to identify good preceptors. Specifically, we identified a key person (usually a physician) within each potential site. This liaison then informed us whether the site was suitable for ICE, with the main criterion being that the site had a variety of professionals with whom students could work. The liaison also gauged capacity (the number of students that could rotate through the clinic at any given time), provided names and roles of preceptors, and informed preceptors of the goals of ICE. When scheduling students, we followed up directly with preceptors, again sharing ICE learning objectives and what they could expect.

A potential limitation of the clinical introduction session may be recruiting eight to nine professionals to serve as station leaders. If recruiting eight or nine professionals is not possible, consider recruiting more than one from each category or reducing the number of stations.

Pedagogical challenges included providing quality feedback on student reflections, as not all faculty had prior training in fostering reflective capacity. Faculty should provide feedback for both content and reflective skill, as this produces the most learning. In the What? So What? Now What? model of reflection, this would mean that faculty acknowledge what the student has written, whether the student has explored the significance of what he or she has described, and what, if anything, the student has written about utilizing the observations or gaining new knowledge as a result of them. We also recommend faculty development in fostering reflective capacity. This might include further exploration of the What? So What? Now What? model of reflection, workshopping feedback with other faculty, and introducing faculty to other reflection-grading tools such as the REFLECT (Reflection Evaluation for Learners’ Enhanced Competencies Tool)** and BEGAN (Brown Educational Guide to the Analysis of Narrative) rubrics.^15,4^

Students also expressed a need for fewer and “more meaningful reflections.” As a result of these comments, the number of required reflections has been reduced by half, and we have provided more specific reflection prompts (Appendix G).

An ongoing challenge for ICE is providing a more active role for students within the health care team. This could be mitigated by scheduling ICE activities in the morning when sites are more active, providing more information about the goals of ICE and preceptor responsibilities to all preceptors, and encouraging students to be more self-directed by completing the prereading and engaging preceptors.

Overall, we achieved our goal of introducing first-year medical students to health care teams using interprofessional education and experiential learning. This model allows students to learn aspects of team-based health care early in their medical career.

## Appendices

A. ICE Instructor Packet.docx
B. Prequiz.docx
C. Clinical Introduction Session.docx
D. Instructions for Video in Clinical Introduction.docx
E. Video in Clinical Introduction Session.mp4
F. ICE Reading List.docx
G. Reflection Assignment Instructions.docx
H. Guide on How to Reflect.docx
I. Experience and Reflection Notes.docx
J. Small-Group Debriefing and Guiding Questions.docx
K. Fall Semester Term Paper Instructions.docx
L. Winter Semester Term Paper Instructions.docx
M. Sample Preceptor Assessment Form.docx
N. Sample Course Evaluation Form.docx All appendices are peer reviewed as integral parts of the Original Publication.
