# Supplementary material for: The Interprofessional Clinical Experience: Introduction to Interprofessional Education Through Early Immersion in Health Care Teams
Source: MedEdPORTAL. 2017 Mar 30;13:10564. doi: 10.15766/mep_2374-8265.10564 (PMC6342292; doi:10.15766/mep_2374-8265.10564)
Supplement: Supplementary file 1 — A. ICE Instructor Packet.docx B. Prequiz.docx C. Clinical Introduction Session.docx D. Instructions for Video in Clinical Introduction.docx E. Video in Clinical Introduction Session.mp4 F. ICE Reading List.docx G. Reflection Assignment Instructions.docx H. Guide on How to Reflect.docx I. Experience and Reflection Notes.docx J. Small-Group Debriefing and Guiding Questions.docx K. Fall Semester Term Paper Instructions.docx L. Winter Semester Term Paper Instructions.docx M. Sample Preceptor Assessment Form.docx N. Sample Course Evaluation Form.docx [file mep-13-10564-s001.zip › A. ICE Instructor Packet.docx]

**Interprofessional Clinical Experience (ICE) Instructor’s Guide**

This packet provides comprehensive instructions for using materials related to the Interprofessional Clinical Experience (ICE) course. ICE is a year-long course designed to introduce pre-clinical students to team-based aspects of the healthcare environment and provide them with a foundation upon which later experiences can grow. The ability to collaborate as a member of interprofessional teams is essential for patient care and a core competency for students in health professions education. The course is divided into two parts: one half-day orientation and biweekly longitudinal experiences with a variety of healthcare professionals in inpatient and outpatient settings. Based on principles of experiential learning (e.g., combining experience with theory; engaging in purposeful endeavors; encouraging systems-based thinking through interaction with complex systems, incorporating reflection, etc.^[[1]](#endnote-1)^) and interprofessional education (e.g., experiences where practitioners and students learn from each other in an academic or workplace-based settings^[[2]](#endnote-2)^), ICE introduces students to interprofessional practice, systems-based medical practices, and varying compositions of healthcare teams.

**Educational Objectives:**

By the end of the course, students will be able to:

1. Describe the roles and responsibilities of a variety of healthcare professionals;
2. Utilize effective communication with other health professionals on healthcare teams;
3. Develop an understanding of the basic organization of healthcare systems;
4. Examine their own and others’ perspectives by engaging in self-directed learning and reflective practice.

**Course Materials Included in this Packet:**

**Introduction to the Interprofessional Clinical Experience (ICE) & Interprofessional Education (IPE)**

Appendix B: Pre-quiz

Appendix C: Clinical Introduction Session

Appendix D: Instructions for Video in Clinical Introduction Session

Appendix E: Video in Clinical Introduction Session

The pre-quiz measures students’ baseline knowledge of various healthcare professionals and can be administered electronically either a couple of days before the Clinical Introduction Session or as a warm-up activity during the Clinical Introduction Session. Students going into medicine may assume that they know the roles and responsibilities of other professionals; this instrument helps to identify areas of knowledge gaps and to prime students to important areas of learning.

The Clinical Introduction Session is a half-day (approximately four hours) session that provides students an initial overview of some of the roles they will encounter during their longitudinal experience. In a large group setting, students view the Video for Clinical Introduction Session. Then in small groups (10-12 students), students rotate through multiple stations led by different health professionals for 15-minute intervals, learning about the professionals’ background and roles/responsibilities within the healthcare team. An active learning experience, this introductory session allows students to utilize prior knowledge, ask questions, and develop new understanding.

**Site Visit Preparation**

Appendix F: Interprofessional Clinical Experience (ICE) Reading List

The ICE Reading List offers students context and background information on the various health professions they will encounter during their longitudinal experience. Readings also help them be more actively engaged during the experience. Students should complete readings relevant to the professional they will shadow prior to each site visit.

**Post-Visit Activities**

Appendix G: Reflection Assignment Instructions

Appendix H: How to Reflect

Appendix I: Experience & Reflection Notes

Appendix J: Small Group Debriefing Lesson Plan and Guiding Questions

Students document and critically reflect on their experiences through reflective writing. Reflection Assignment Instructions and How to Reflect should be distributed in advance of their first site visit. The Experience and Reflection Notes handout can guide student note-taking while on site.

An hour-long small group (10-12 students) debriefing session should take place twice per semester. Led by faculty small group facilitators, these sessions trigger critical reflection around important themes such as medical errors, decision-making, and challenges to interprofessional practice. They also allow students to connect with each other, describe and analyze their experiences, find commonalities and differences, and address any questions or concerns that may have come up during the experience.

**Course Assignments**

Appendix K: Fall Semester Term Paper Instructions

Appendix L: Winter Semester Term Paper Instructions

End-of-term papers encourage deep introspection and synthesis of experiences. In ICE, there are two such papers requiring students to consider their experiences as a whole and write about how their awareness of self and others as medical professionals changed over the course of the term; what trends in communication, teamwork, or systems they observed; and what they hope to learn moving forward. Students can also reflect on how their understanding of themselves as professionals have evolved over time and what impact the experience had on their professional development.

**Evaluations/Assessments**

Appendix M: Sample Preceptor Assessment Form

Appendix N: Sample Course Evaluation Form

After each ICE experience, students should seek feedback from the professional they observed. This assessment will evaluate them on their professionalism, communication skills, and teamwork.

Students should evaluate ICE using a form such as the sample course evaluation form at the end of each term.

1. Proudman, B. (1992). Experiential education as emotionally-engaged learning. *The Journal of Experiential Education 15*(2), 19-23. [↑](#endnote-ref-1)
2. Center for the advancement of Interprofessional Education (2002). Defining IPE. Accessed from <http://caipe.org.uk/resources/defining-ipe/> [↑](#endnote-ref-2)
