# Supplementary material for: The Interprofessional Clinical Experience: Introduction to Interprofessional Education Through Early Immersion in Health Care Teams
Source: MedEdPORTAL. 2017 Mar 30;13:10564. doi: 10.15766/mep_2374-8265.10564 (PMC6342292; doi:10.15766/mep_2374-8265.10564)
Supplement: Supplementary file 1 — A. ICE Instructor Packet.docx B. Prequiz.docx C. Clinical Introduction Session.docx D. Instructions for Video in Clinical Introduction.docx E. Video in Clinical Introduction Session.mp4 F. ICE Reading List.docx G. Reflection Assignment Instructions.docx H. Guide on How to Reflect.docx I. Experience and Reflection Notes.docx J. Small-Group Debriefing and Guiding Questions.docx K. Fall Semester Term Paper Instructions.docx L. Winter Semester Term Paper Instructions.docx M. Sample Preceptor Assessment Form.docx N. Sample Course Evaluation Form.docx [file mep-13-10564-s001.zip › B. Prequiz.docx]

**Appendix B: Pre-Quiz**

**Faculty Instructions:** This quiz can be administered electronically either a couple of days before the Clinical Introduction Session or as a warm-up activity during the Clinical Introduction Session. It should not be graded, as the purpose is to provide a baseline measure of students’ knowledge of various healthcare professionals. If administered before the Clinical Introduction Session, the aggregate results can be shared with the various professionals and used as a point of discussion. Answer key can be found at the end of the quiz.

**Student Instructions:** Please answer the following questions:

1. In which of the following hospital settings are child life specialists most likely to work?
2. Emergency Department
3. Neonatal Intensive Care Unit
4. Pediatric Intensive Care Unit
5. Obstetrics/Delivery
6. Daycare Facility
7. Patients need to have appropriate nutrition to promote healing. Who has college-level training to help patients eat appropriately in the hospital?
   1. Nutritionists
   2. Clerks
   3. Medical assistants
   4. Dieticians
   5. Meal Planners
8. All of the following are true of the physical therapy profession, except:
9. Physical therapists typically have a doctoral degree
10. Physical therapists work with patients to clear mucous out of their lungs
11. Physical therapists can write prescriptions
12. Physical therapists exercise and walk with patients who are on a ventilator
13. Physical therapists develop and implement discharge plans
14. Which is correct about the work of physician assistants?
    1. Physician assistants can perform surgery independently
    2. Physician assistants can work in the emergency department
    3. Physician assistants can prescribe narcotics without supervision
    4. Physician assistants can intubate on their own
    5. Physician assistants can perform lumbar punctures unsupervised
15. Which of the following tasks can a pharmacist perform with additional training?
16. Check blood pressure
17. Give immunizations
18. Prescribe medications
19. Assist in codes
20. All of the above
21. Respiratory therapists can do all of the following except:
22. Manage ventilator settings during air medical transport
23. Intubate a patient in the operating room
24. Alter settings on BIPAP / CPAP machines in the home
25. Prescribe inhalers for COPD
26. Educate students in elementary schools on asthma management
27. The primary role of inpatient medical social workers is:
28. Helping patients file Medicaid applications
29. Providing patients with support and information about psychosocial needs
30. Assisting patients with lodging and parking passes
31. Assisting patients with nursing home placements
32. Providing child care for parents who are patients
33. Medical Assistants contribute to health care. What are the tasks of a Medical Assistant?
34. Drawing blood for lab tests
35. Measuring patients’ vital signs
36. Arranging for hospital admissions
37. Coding and filling out insurance forms
38. All of the above
39. Occupational therapists’ roles in patient care may include:
40. Helping patients find work that fits their physical abilities
41. Teaching mobility strategies to facilitate independence
42. Deciding if a patient is physically fit to return to work
43. Coordinating workman’s compensation
44. Exercising with a patient on a daily basis
45. Which of the following is true regarding pharmacists training and practice?
46. All pharmacists need a Doctor of Pharmacy degree to be licensed to practice
47. Pharmacists can receive specialty board-certification in internal medicine
48. Pharmacists must complete a residency to practice in retail pharmacy
49. Pharmacists must obtain a federal drug enforcement agency (DEA) number
50. Pharmacists may change drug dosages without consulting the provider
51. Which of the following is within the scope of social work in a medical setting?
52. Filing for additional insurance to cover hospital bills
53. Prosecuting for abuse and neglect of an elderly patient
54. Removing children from unsafe living situations
55. Assisting with communication between families and medical providers
56. Conducting a home safety assessment
57. Nurses require specific education. Which of the following is true of their training?
58. Registered nurses can specialize in specific areas such as ambulatory care
59. Registered nurses can be educated in a 4-year bachelors program
60. Registered nurses can be educated in 2-year associate nursing program
61. Registered nurses may have an Associate Degree in Nursing or a Bachelor of Science in Nursing
62. All of the above
63. Which of the following duties can child life specialists perform?
    1. Distract a child during a procedure
    2. Counsel families regarding educational services for children with disabilities
    3. Ensure the home environment is safe for a child
    4. Advise families on how to discipline a child
    5. Examine a child for evidence of child abuse
64. Many health care professionals need to be licensed. Which of the following professions do not need a license?
    1. Dentist
    2. Speech and Language Pathologist
    3. Chaplain
    4. Pharmacist
    5. Social Worker
    6. X-ray / Radiation Technician
65. Which professional can start IV’s in the hospital?
    1. EMT/ Paramedic
    2. Recreational Therapist
    3. Rehabilitation Engineers
    4. Speech and Language Pathologist
    5. Clerk
66. A hospital chaplain’s role is to
    1. Hold Catholic mass at the hospital
    2. Provide counseling and guidance to patients of any faith
    3. Administer last rites to Catholics
    4. Discuss and document code-status with patients
    5. Counsel Jehovah’s witnesses on refusal of blood products
67. Who can take a chest X-ray of a patient?
    1. A radiologist
    2. An ultrasound technologist
    3. A radiology technologist
    4. A physician
    5. A nurse
68. Which professional cannot perform procedures?
    1. Doctor
    2. Dentist
    3. Physician Assistant
    4. Radiologist
    5. Geneticist

Pre-Quiz Answer Key:

| 1. a  2. d  3. c  4. b  5. e  6. d | 7. b  8. e  9. b 10. a  11. d  12. e | 13. a  14. c  15. a  16. b  17. c  18. e |
| --- | --- | --- |
