# Supplementary material for: The Interprofessional Clinical Experience: Introduction to Interprofessional Education Through Early Immersion in Health Care Teams
Source: MedEdPORTAL. 2017 Mar 30;13:10564. doi: 10.15766/mep_2374-8265.10564 (PMC6342292; doi:10.15766/mep_2374-8265.10564)
Supplement: Supplementary file 1 — A. ICE Instructor Packet.docx B. Prequiz.docx C. Clinical Introduction Session.docx D. Instructions for Video in Clinical Introduction.docx E. Video in Clinical Introduction Session.mp4 F. ICE Reading List.docx G. Reflection Assignment Instructions.docx H. Guide on How to Reflect.docx I. Experience and Reflection Notes.docx J. Small-Group Debriefing and Guiding Questions.docx K. Fall Semester Term Paper Instructions.docx L. Winter Semester Term Paper Instructions.docx M. Sample Preceptor Assessment Form.docx N. Sample Course Evaluation Form.docx [file mep-13-10564-s001.zip › C. Clinical Introduction Session.docx]

**Appendix C: Clinical Introduction Session**

The Clinical Introduction Session provides students with an initial overview of some of the roles they will encounter during their longitudinal experience. It involves reviewing a short video of a clinical case (Appendices D-E) that demonstrates how various members of the healthcare team are involved in patient care, followed by small groups (10-12 students). In the small groups, students rotate through multiple stations at 15-minute intervals. Each station is led by a different health professional, such as nurses, social workers, medical assistants, etc. Each of these professionals provides students with key background about their profession and answers questions for a deeper understanding of their roles and responsibilities. An active learning experience, this introductory session allows students to utilize prior knowledge, ask questions, and develop new understanding.

**Learning Objectives:**

As a result of this session, students will be able to:

- Increase their understanding of the education and training required for other health professions
- Increase their understanding of the roles other health professionals pay in patient care

**Lesson Plan**

**Pre-Session**

Recruit 8-9 different professionals to lead small group stations. The ideal list of health professions includes: child life specialist, clinical social worker, dietitian, medical assistant, nurse, paramedic, pharmacist, physician’s assistant, and radiologist. However, recruiting such a variety of professionals may not be possible in all settings. In such cases, consider recruiting more than one from each category (e.g., two nurses), or reducing the number of stations.

Inform the recruited professionals that the purpose of the session is to increase student understanding of the education and training required for their health profession and the role their profession plays in patient care. Share the session plan with them, and instruct the professionals to prepare a short, 7-10-minute presentation on their professional background, education, training, and role in patient care. Tell them they may also discuss what role they play in teams.

**Session Plan**

Introduction (5 minutes)

Introduce the session by stating that the practice of medicine is a team activity. High functioning teams know the background of other team members and their role on the team, and effective collaboration requires demonstrated respect for other professions, effective communication, and understanding of common goals. Share the learning objectives of the session, which is to increase students’ understanding of the education and training required for other health professionals they will encounter in the clinics and what these professionals’ roles are in patient care. Then briefly describe how the session will run: they will watch a video demonstrating how patient care is delivered in teams from the patient perspective and then break into small groups and rotate through 8-9 stations in 15-minute intervals in order to learn about other members of the care team.

Activity 1: Video (30-35 minutes; large group)

The purpose of the video (Appendix D) is to demonstrate how different health professionals work together to deliver care. Ask students to take note of what the patient and the parent share about their experience and what advice they offer to physicians. Tell them they will also hear from a physician and to take note of the advice offered by the physician. After screening the video, ask students to share their observations or insights with the group. Focus the discussion further by asking questions such as, “What does effective collaboration look like?” and “What are some tips for communicating clearly and effectively with other professionals in the healthcare team? With the patient? With the parent?”

Activity 2: Small Groups (2 – 2 ½ hours)

While in the large group, tell students that they will be divided into groups of 10-12 students (this can be done in advance or you can use existing small group assignments). Each group will start with a different professional and rotate through all stations in 15-minute intervals. During the first 10 minutes, the professional at their station will share their professional background, education, training, and role in patient care. The various professionals may also talk about what the team experience is like for them personally. Then students will have 5 minutes to ask questions. Once the 15 minutes are up, they will move on to the next station and repeat the process.

Activity 3: Large Group Closing (10 minutes)

After the students have rotated through all of the stations, gather together in the large group. Ask students to share any insights they gained from talking with various professionals.
