# Supplementary material for: The Interprofessional Clinical Experience: Introduction to Interprofessional Education Through Early Immersion in Health Care Teams
Source: MedEdPORTAL. 2017 Mar 30;13:10564. doi: 10.15766/mep_2374-8265.10564 (PMC6342292; doi:10.15766/mep_2374-8265.10564)
Supplement: Supplementary file 1 — A. ICE Instructor Packet.docx B. Prequiz.docx C. Clinical Introduction Session.docx D. Instructions for Video in Clinical Introduction.docx E. Video in Clinical Introduction Session.mp4 F. ICE Reading List.docx G. Reflection Assignment Instructions.docx H. Guide on How to Reflect.docx I. Experience and Reflection Notes.docx J. Small-Group Debriefing and Guiding Questions.docx K. Fall Semester Term Paper Instructions.docx L. Winter Semester Term Paper Instructions.docx M. Sample Preceptor Assessment Form.docx N. Sample Course Evaluation Form.docx [file mep-13-10564-s001.zip › D. Instructions for Video in Clinical Introduction.docx]

**Appendix C: Instructions for Video in Clinical Introduction**

**Faculty Instructions:** Use this video (Appendix E – Video in Clinical Introduction.m4v) as part of the Clinical Introduction Session (Appendix C). The video simulates a Pediatric Emergency Room interaction between a chronically ill patient, her mother, and a physician. It’s followed by an interview with the patient and her mother and an interview with a physician, where they discuss effective patient care and teamwork. The video can be used to stimulate discussion about patient-centered care, communication, and teamwork.

Prior to screening the video, mention that while efforts have been made to portray a realistic patient interview, there are some flaws within the video. Namely, the doctor and the medical students are *not* taking notes when the patient, Lily, and her mother are speaking. Remind students that, in reality, they should always take notes when interviewing patients and should never rely on memory alone.

After screening the video, ask students to share any observations or insights gained from the video. Focus the discussion by asking questions such as, “What does effective collaboration look like?” and “What are some tips for communicating clearly and effectively with other professionals in the healthcare team? With the patient? With the parent?”
