# Supplementary material for: The Interprofessional Clinical Experience: Introduction to Interprofessional Education Through Early Immersion in Health Care Teams
Source: MedEdPORTAL. 2017 Mar 30;13:10564. doi: 10.15766/mep_2374-8265.10564 (PMC6342292; doi:10.15766/mep_2374-8265.10564)
Supplement: Supplementary file 1 — A. ICE Instructor Packet.docx B. Prequiz.docx C. Clinical Introduction Session.docx D. Instructions for Video in Clinical Introduction.docx E. Video in Clinical Introduction Session.mp4 F. ICE Reading List.docx G. Reflection Assignment Instructions.docx H. Guide on How to Reflect.docx I. Experience and Reflection Notes.docx J. Small-Group Debriefing and Guiding Questions.docx K. Fall Semester Term Paper Instructions.docx L. Winter Semester Term Paper Instructions.docx M. Sample Preceptor Assessment Form.docx N. Sample Course Evaluation Form.docx [file mep-13-10564-s001.zip › F. ICE Reading List.docx]

**Appendix F: Interprofessional Clinical Experience Reading List**

**Faculty Instructions:** Make all readings available to students prior to the first session. Instruct students to read at least one article about the health profession they’ll shadow before their experience.

**Student Instructions:** The readings for this course have been intentionally selected to provide you with context and background information on each of the health professions you will observe. They will also elaborate on roles, responsibilities, interprofessional teamwork, communication, and other related issues. You should read at least one article about the health professional you work with prior to each session in order to improve your experience; however, due to the logistics of some clinics, you may not know who you will be working with until after you arrive at the clinic. In such cases, read about the health profession after your experience and before you write your reflection.

The U.S. Bureau of Labor Statistics is a good resource for specific information on health professions such as prevalence, salary, and education requirements. Consider searching for job titles on their website in addition to the readings provided.

# **Call Center / Clerk**

Oh J. (2010, October 5). Leadership & management: 6 best practices for improving hospital call efficiency. Becker’s Hospital Review. Retrieved from:<http://www.beckershospitalreview.com/hospital-management-administration/6-best-practices-for-improving-hospital-call-efficiency.html>

Strother JB. (2006). Call centers in health care: Effect on patient satisfaction. *IEEE:* 291-298*.* doi: 10.1109/IPCC.2006.320362

# **Chaplain**

Cramer EM and Tenzek KE. (2012). The chaplain profession from the employer perspective: an analysis of hospice chaplain job advertisements. *Journal of Health Care Chaplaincy, 18*(3-4): 133-150.

Fitchett G, Lyndes KA, Cadge W, Berlinger N, Flanagan E, and Misasi J. (2011). The role of professional chaplains on pediatric palliative care teams: perspectives from physicians and chaplains. *Journal of Palliative Medicine*, 14(6): 704-707. doi: 10.1089/jpm.2010.0523

Gauger RW. (2013). The “un”doing of a hospital chaplain. *Journal of Pastoral Care & Counselling, 67*(3-4).

Stouter DK, Wallace A, Duffy J, Rashid A, and Valentine A. (2012). Long coats, short coats and no coats: chaplaincy presents to psychiatry at the University of Texas MD Anderson Cancer Center, a report. *Journal of Pastoral Care & Counselling*, 66(1): 6.

# **Child Life Specialist**

American Academy of Pediatrics Committee on Hospital Care and Child Life Council. (2014). Policy statement: Child life services. *Pediatrics, 133*(5): e1471-e1478

Crider J and Pate MF. (2011). Helping children say goodbye to loved ones in adult and pediatric units: certified child life specialist--critical care nurse partnership. *AACN Advanced Critical Care Nursing, 22*(2): 109-112. doi: 10.1097/NCI.0b013e31820810b6

Rubin S. (1992). What’s in a name? Child life and the play lady legacy. *Children’s Health Care, 21*(1): 4-13.

Turner JC and Fralic J. (2009). Making explicit the implicit: Child life specialists talk about their assessment process. *Child & Youth Care Forum, 38*(1): 39-54. doi: 10.1007/s10566-009-9066-x

Wright, J. (2014). Sweet success: Lessons from a child life specialist. *Nursing, 44*(2): 30.

# **Clinical Social Worker**

Gregorian C. (2005). A career in hospital social work: Do you have what it takes? *Social Work in Health Care, 40*(3): 1-14.

Reamer FG. (2014, November). Prima facie and actual moral duties in social work. *Social Work Today.*

# **Dentist**

American Dental Association. (2015). *Education and Careers.* Retrieved from:<http://www.ada.org/en/education-careers>

Mouradian WE, Lewis CL, and Berg JH. (2014) Integration of dentistry and medicine and the dentist of the future: The need for the health care team. *Journal of the California Dental Association, 42*(10): 687-696.

Whitney C. (2014). How dentists can collaborate with physicians and bridge the oral-systemic gap. *Dental Practice Management*. Retrieved from:<http://practicemanagement.dentalproductsreport.com/management-oral-systemic-link/how-dentists-can-collaborate-physicians-bridge-oral-systemic-gap>

# **Dietitians**

DeLegge MH, and Kelly AT. (2013). State of nutrition support teams. Nutrition in Clinical Practice, 28(6): 691-697.

Flesher M, Kinloch K, Grenon E and Coleman J. (2011). Access to dietitians in primary health care. *Canadian Journal of Dietetic Practice & Research, 72*(1): 32-36.

Jortberg, B, Fleming, M. (2014). Registered dietitian nutritionists bring value to emerging health care delivery models. *Journal of the Academy of Nutrition and Dietetics*, *114*(12): 2017-2022.

Van Beek K, Duchemin S, Gersh G, Pettigrew S, Silva P, and Luskin B. (2008). Counseling and wellness services integrated with primary care: a delivery system that works. *The Permanente Journal, 12*(4): 20-24.

**EMTs / Paramedics**

Paris PM and O’Connor RE. (2008). A national center for EMS provider and patient safety: Helping EMS providers help us. *Prehospital Emergency Care, 12*(1): 92-94.

Schwartz B. (2015). Transfer of care and offload delay: continued resistance or integrative thinking? *Canadian Journal of Emergency Medicine*.

**Geneticist**

American Board of Medical Genetics and Genomics. Specialties of Genetics. Retrieved from <http://www.abmgg.org/pages/training_specialties.shtml>

# **Medical Assistant**

Elder NC, Jacobson CJ, Bolon SK, Fixler J, Pallerla H, Busick C, Gerrety E, Kinney D, Regan S, and Pugnale M. (2014). Patterns of relating between physicians and medical assistants in small family medicine offices. *Ann Fam Med, 12*(2): 150-157.

Taché S and Chapman S. (2005). What a medical assistant can do for your practice. *Family Practice Management, 12*(4): 51-54.

# **Nurse**

Battie R and Steelman VM. (2014). Accountability in nursing practice: Why it is important for patient safety. *AORN Journal, 100*(5): 537-541.

Nursing roles in the spotlight: panelists discuss iom's recommendations for new practice models. *Modern Healthcare* [serial online]. 2011 Jan 3 3, 2011;41(1):23.

Secemsky, B. (2012, November 9). Doctors and nurses: a relationship in the works. *The Huffington Post*.

# **Pharmacists**

Chui MA, Stone JA, Odukoya OK, and Maxwell L. (2014), Facilitating collaboration between pharmacists and physicians using an iterative interview process. *Journal of the American Pharmacists Association,* *54*(1): 35-41.

McCullough MB, Petrakis BA, Gillespie C, Solomon JL, Park AM, Ourth H, Morreale A, and Rose AJ. (2015), Knowing the patient: A qualitative study on care-taking and the clinical pharmacist-patient relationship. *Research in Social and Administrative Pharmacy,*

# **Physical Therapists/Occupational Therapists**

Reis E. (2014). The advocate in aggregate. *PT in Motion, 6*(6): 16-21.

Torres A, Kunishige N, Morimoto D, Hanzawa T, Ebesu M, Fernandez J, Nohara L, SanAgustin E, and Borg S. (2015). Shared governance: A way to improve the care in an inpatient rehabilitation facility. *Rehabilitation Nursing, 40*(2): 69-73.

# **Physician’s Assistant**

Frisch, S. (2015) The physician assistant will see you now. *Minnesota Medicine, 98*(2): 8.

Loxterkamp D. (2014) What a doctor is good for. *BMJ 349:* g6894.

**Radiologist**

Margolies L, Salvatore M, Eber C, Jacobi A, Lee I, Liang M, Tang W, Xu D, Zhao S, Minal K, Wisnivesky J, Henschke C, Yankelevitz D. (2015). The general radiologist’s role in breast cnacer risk assessment: breast density measurement on chest CT. *Clinical Imaging 39(6):*979-982.

**Radiology Technologist**

Who are radiologic technologists? Retrieved from <https://www.asrt.org/main/careers/careers-in-radiologic-technology/who-are-radiologic-technologists>

**Recreational Therapist**

What is RT/TR? Retrieved from <https://www.atra-online.com/what/FAQ>

# **Rehabilitation Engineers**

National Institute of Biomedical Imaging and Bioengineering. (n.d.). *Rehabilitation Engineering*. Retrieved from:<http://www.nibib.nih.gov/science-education/science-topics/rehabilitation-engineering>

Chau T, Moghimi S, and Popovic MR. (2013). Knowledge translation in rehabilitation engineering research and development: A knowledge ecosystem framework. *Archives of Physical Medicine and Rehabilitation, 94*(1): S9-S19. doi: 10.1016/j.apmr.2012.07.032

# **Speech and Language Pathologist**

# Giles M, Barker M, and Hayes A. (2014). The role of the speech-language pathologist in home care. *Home Healthcare Nurse, 32*(6): 349-353.

# Radtke JV, Baumann BM, Garrett KL, and Happ MB. (2011) Listening to the voiceless patient: case reports in assisted communication in the intensive care unit. *Journal of Palliative Medicine, 14*(6):791-795.

Wertheimer JC, Roebuck-Spencer TM, Constantinidou F, Turkstra L, Pavol M, and Paul D. (2008). Collaboration between neuropsychologists and speech-language pathologists in rehabilitation settings. *Journal of Head Trauma Rehabilitation, 23*(5): 273-285.

# **Ultrasound Technologist**

Burton, E. (2016) Guide to becoming an ultrasound technologist. *Careers*. Retrieved from <http://www.innerbody.com/careers-in-health/guide-to-becoming-ultrasound-technologist.html>

# **X-Ray / Radiology Technician**

The American Registry of Radiologic Technologists. (2015). *Radiologic Technologists.*

Jacobs BR, Crotty E, Conway E, Hart KW, Dietrich C, Pettinichi S, and Racadio J. (2010). Computerized Provider Order Entry with Pager Notification Improves Efficiency in STAT Radiographic Studies and Respiratory Treatments. *Applied Clinical Informatics, 1*(1): 19-31.

Poling CC. (2014). R.T.s Tame the “Tiger in the Room.” *ASRT Scanner 46*(4): 26-31.
