# Supplementary material for: The Interprofessional Clinical Experience: Introduction to Interprofessional Education Through Early Immersion in Health Care Teams
Source: MedEdPORTAL. 2017 Mar 30;13:10564. doi: 10.15766/mep_2374-8265.10564 (PMC6342292; doi:10.15766/mep_2374-8265.10564)
Supplement: Supplementary file 1 — A. ICE Instructor Packet.docx B. Prequiz.docx C. Clinical Introduction Session.docx D. Instructions for Video in Clinical Introduction.docx E. Video in Clinical Introduction Session.mp4 F. ICE Reading List.docx G. Reflection Assignment Instructions.docx H. Guide on How to Reflect.docx I. Experience and Reflection Notes.docx J. Small-Group Debriefing and Guiding Questions.docx K. Fall Semester Term Paper Instructions.docx L. Winter Semester Term Paper Instructions.docx M. Sample Preceptor Assessment Form.docx N. Sample Course Evaluation Form.docx [file mep-13-10564-s001.zip › G. Reflection Assignment Instructions.docx]

**Appendix G: Reflection Assignment Instructions**

Over the course of the academic year, students submit six individual reflections of approximately 400 words each (3 in the fall and 3 in the winter semester). The Course Director is the intended audience for these reflections.

**Student Instructions:**

- Before the clinical experience, familiarize yourself with the role you will be observing by completing the recommended readings (you **must** read at least one article).
- During the experience, it will be helpful to complete the reflection handout (Appendix I)
- Select one of the questions below
- Using your notes from the reflection handout, and any notes you have from the readings, compose your reflection using the “What? So What? Now What?” model of reflection (Appendix H)

**Reflection Questions:**

1. Reflect on your initial experience at your ICE placement. What, if anything, in your observations surprised or intrigued you? How did the experience compare with any of your prior clinical experience? Did you have any preconceived notions about this or other clinical settings prior to this experience? If so, what were they and how were they affected by this experience?
2. Self-directed learning, in its broadest meaning, describes a process in which individuals take the initiative with or without the help of others, in diagnosing their learning needs, formulating learning goals, identifying resources for learning, choosing and implementing learning strategies, and evaluating learning outcomes. Taking this into consideration, please reflect on the following:
   1. What do I hope to learn?
   2. What did I observe about topics I learned in my medical education (e.g., aspects of confidentiality)?
   3. Are there any clinical/scientific questions that I am curious about and can investigate? How can I find out more?
   4. What more can I know/learn? How?
3. Reflect on the implementation of patient-centered approaches to medical care at your site. In your opinion, how well does your site implement patient-centered practices, if at all? How do patients respond to these methods? How do you think providers feel about these approaches, and how do you know? If you think a patient-centered approach is lacking at your site, what are some areas for improvement?
4. Reflect on the ways that members of the healthcare team at your ICE site communicate with one another and with patients. Are there specific tools or protocols that they rely on? In what ways do you see effective or ineffective communication between team members and/or patients influencing quality of care? Are there areas for improvement here?
5. Reflect on the degree to which you have observed the impact of social determinants of health (socioeconomic status, education, race, etc.) in your ICE experience. To what degree do providers – either individually or as part of a broader team or system – assess and work to address potential barriers that their patients may face?
6. Unconscious bias refers to a bias that we are unaware of and which happens outside of our control. It is a bias that happens automatically and is triggered by our brain making quick judgments and assessments of people and situations, influenced by our background, cultural environments, and personal experiences. Were there any assumptions made or unconscious bias at play in the care provided to patients at your site? What were some of the assumptions you had about this patient? Why?
7. Reflect on areas of “waste” in the clinic. This can be people or patient’s time, material, providing non-visit related services, waiting for resources to become available, or other areas. How could the clinic be more efficient?
8. Did you witness any preventable adverse events or near misses affecting patients; what factors in the way the system of care delivery is designed (environment, communication, technology, teams, etc.) contributed to the adverse event or near miss; what types of changes could reduce the likelihood of this happening in the future? Did witnessing an error or near miss affect your view of the individual or that professional in general?
9. If you are unable to identify any of the suggested reflection topics above, please write your own question and answer it.
