# Supplementary material for: The Interprofessional Clinical Experience: Introduction to Interprofessional Education Through Early Immersion in Health Care Teams
Source: MedEdPORTAL. 2017 Mar 30;13:10564. doi: 10.15766/mep_2374-8265.10564 (PMC6342292; doi:10.15766/mep_2374-8265.10564)
Supplement: Supplementary file 1 — A. ICE Instructor Packet.docx B. Prequiz.docx C. Clinical Introduction Session.docx D. Instructions for Video in Clinical Introduction.docx E. Video in Clinical Introduction Session.mp4 F. ICE Reading List.docx G. Reflection Assignment Instructions.docx H. Guide on How to Reflect.docx I. Experience and Reflection Notes.docx J. Small-Group Debriefing and Guiding Questions.docx K. Fall Semester Term Paper Instructions.docx L. Winter Semester Term Paper Instructions.docx M. Sample Preceptor Assessment Form.docx N. Sample Course Evaluation Form.docx [file mep-13-10564-s001.zip › H. Guide on How to Reflect.docx]

**Appendix H: Guide on How to Reflect**

**Faculty Instructions:** “How to Reflect” walks students through some of the basics of reflection. Share this document with students before their first reflection assignment is due.

**What is reflection?**

Reflection is the “process where we look at an experience, frame it, and derive meaning from it.”^^[[1]](#endnote-1)^^ It usually requires you to list, describe, or log an experience. In this course, you will be asked to not only reflect on your clinical experience, but also to think critically about each experience. That means you will need to question, compare, contrast, or critique your experience. You may go further by identifying conflict you observe and offering solutions.

**What does it mean to be a reflective professional?**

Reflective professionals are those who:

- have an open mind
- think about their own thinking
- are willing to deepen learning
- acknowledge and connect with their feelings
- consider others’ perspectives
- acknowledge errors, problems, and learning gaps, and seek to learn from them
- create plans to direct future action based on what they have learned

Anyone can become a reflective professional by developing their skills in critical reflection through ongoing practice and feedback. This course is designed to help you cultivate these skills.

**Why reflect?**

There are many benefits to reflecting critically, and an increasing number of studies are supporting this theory. For health professional, critical reflection improves:

- clinical reasoning, problem-solving, and self-assessment skills
- test scores
- diagnostic accuracy
- interactions with standardized patients
- intangible attributes such as empathy, humanism, professionalism, and self-awareness
- management of complex health systems and patients
- self-care

**What do I reflect on?**

Over the course of the year, you’ll be asked to write a number of reflections. The audience for these reflections are ICE faculty. For these reflections, you should do the following:

- Before the clinical experience, familiarize yourself with the role you will be observing by completing the recommended readings
- During the experience, it will be helpful to complete the Experience & Reflection Notes handout (Appendix I)
- Select one of the questions in the reflection assignment (Appendix G)
- Using your notes from the reflection handout, and any notes you have from the readings, compose your reflection using the “What? So What? Now What?” model of reflection, also described below

**How do I reflect?**

All reflections require observation, introspection, and articulation. As such, begin by jotting down notes about the event (e.g., conversations, activities, thoughts, feelings). Then, engage in introspection by making time to create meaning of your observations. Finally, articulate these in writing.

You can do the above by using the “What? So What? Now What?” model of reflection. This memorable model asks you to describe an event or some observations, analyze or evaluate your description, and synthesize the information. The process is elaborated in the table below:

| **What?** | **So What?** | **Now What?** |
| --- | --- | --- |
| Description/Self-Awareness | Analysis/Evaluation | Synthesis |
| Example questions:  What happened?  What did I do? What did others do?  What was I trying to achieve?  What was good or bad about the experience?  What does this remind me of? | Example questions:  So what is the importance of this?  So what more do I need to know about this?  So what have I learned about this? | Example questions:  Now what could I do (i.e., what type of a specific, measurable, attainable, relevant, and timely plan could I make to address similar challenges in the future)?  Now what do I need to do?  Now what might I do?  Now what might be the consequences of this action? |

**How will reflections be graded?**

Reflections will be graded on both content and reflective skill. That means, faculty will provide feedback on the quality of your description (the “What?” in the reflection), your exploration of your observations (the “So What?” in the reflection), and how you plan to utilize or further your learning (the “Now What?” in the reflection).

1. Pagano, M., & Roselle, L. (2009). Beyond reflection through an academic lens: Refraction and international experiential education. *Frontiers: The Interdisciplinary Journal of Study Abroad, 18*, 217-229. [↑](#endnote-ref-1)
