# Supplementary material for: The Interprofessional Clinical Experience: Introduction to Interprofessional Education Through Early Immersion in Health Care Teams
Source: MedEdPORTAL. 2017 Mar 30;13:10564. doi: 10.15766/mep_2374-8265.10564 (PMC6342292; doi:10.15766/mep_2374-8265.10564)
Supplement: Supplementary file 1 — A. ICE Instructor Packet.docx B. Prequiz.docx C. Clinical Introduction Session.docx D. Instructions for Video in Clinical Introduction.docx E. Video in Clinical Introduction Session.mp4 F. ICE Reading List.docx G. Reflection Assignment Instructions.docx H. Guide on How to Reflect.docx I. Experience and Reflection Notes.docx J. Small-Group Debriefing and Guiding Questions.docx K. Fall Semester Term Paper Instructions.docx L. Winter Semester Term Paper Instructions.docx M. Sample Preceptor Assessment Form.docx N. Sample Course Evaluation Form.docx [file mep-13-10564-s001.zip › I. Experience and Reflection Notes.docx]

**Appendix I: Experience & Reflection Notes**

**Faculty Instructions:** The handout below provides guiding questions that help students prepare for and actively observe during their ICE experience. Share it with students prior to their first ICE experience.

**Student Instructions:** Use the handout below to document your ICE experience. The guiding questions will help you take note of important aspects of the experience and can be used to write your reflections.

| **Interprofessional Clinical Experience**  **Experience & Reflection Notes** | **Date / Location** |
| --- | --- |
| ***Preparation***  Before you enter a clinical setting, prepare yourself by asking yourself questions such as:   - What do I know about this profession (e.g., nursing)? What is their role within the health system? What type of preparation have they had for this role? - What experiences, if any, have I had with these professionals? - What is my attitude toward this profession? - How do I feel about actively observing this professional? - What do I hope to learn? |  |
| ***Observation***  During the experience, you can take notes on:   - What are these professionals doing? What can this professional uniquely provide a patient? - What errors can this professional prevent? - What type(s) of communication do they use (e.g., verbal, written, or non-verbal)? How is this different from other professionals in that setting (e.g., doctors)? How does this professional (e.g., nurse) communicate with a doctor vs. a patient vs. significant other(s)? - How do you think this way of communicating with patients/significant other(s) compares with the types of communication you will use as a healthcare professional? - What is the impact of communication on patient care? - When does their training/preparation take effect? - What are the power dynamics between this professional and patients, and between this professional and other professionals? - How does hierarchy or power dynamics affect trust in the workplace? - Are there any conflicts that I am observing? - What am I feeling? Why? - Are there any clinical/scientific questions that I am curious about and can investigate? |  |
| ***Interaction***  You can also ask the other health professionals questions such as:   - Do your observations of them reflect a typical day? - What types of communication does the health/social care professional normally have with patients and what information do they normally give? At what points during the patient’s care do they communicate with patients? - How does the health/social care professional manage situations where patients are angry or upset? What strategies can be adopted to help professionals cope with challenging situations? - Does the healthcare team discuss and agree on the information that is shared with patients/significant other(s) and who will give this information? - With what other professions would the health/social care professional normally communicate? - Would the health/social care professional normally communicate with a member of your healthcare profession and if so, in what situations? |  |
| ***Articulation/Reflection***  After the experience, consider your observations and interactions, and ask yourself:   - What have I learned from this experience? Have my views about this profession changed/stayed the same? - What are some lessons for me to take away from this experience (e.g., if you had to give your future self a set of instructions on how to communicate with these professionals, what advice would you give)? - What did I observe about topics I learned in my medical education (e.g., aspects of confidentiality)? - What more can I know/learn? How? |  |
