# Supplementary material for: The Interprofessional Clinical Experience: Introduction to Interprofessional Education Through Early Immersion in Health Care Teams
Source: MedEdPORTAL. 2017 Mar 30;13:10564. doi: 10.15766/mep_2374-8265.10564 (PMC6342292; doi:10.15766/mep_2374-8265.10564)
Supplement: Supplementary file 1 — A. ICE Instructor Packet.docx B. Prequiz.docx C. Clinical Introduction Session.docx D. Instructions for Video in Clinical Introduction.docx E. Video in Clinical Introduction Session.mp4 F. ICE Reading List.docx G. Reflection Assignment Instructions.docx H. Guide on How to Reflect.docx I. Experience and Reflection Notes.docx J. Small-Group Debriefing and Guiding Questions.docx K. Fall Semester Term Paper Instructions.docx L. Winter Semester Term Paper Instructions.docx M. Sample Preceptor Assessment Form.docx N. Sample Course Evaluation Form.docx [file mep-13-10564-s001.zip › J. Small-Group Debriefing and Guiding Questions.docx]

**Appendix J: Small Group Debriefing Lesson Plan and Guiding Questions**

During this session faculty will guide students in a discussion/debrief of ICE experiences.

**Lesson Plan**

Pre-work:

Share question #1 with students in advance, and ask them to bring their answers to the session.

1. Based on what you’ve observed during the ICE visits so far:
   1. Did you witness any preventable adverse events or near misses affecting patients? What factors in the way the system of care delivery is designed (environment, communication, technology, teams, etc.) contributed to the adverse event or near miss? What types of changes could reduce the likelihood of this happening in the future?

Debriefing Agenda:

1. Have students share where their ICE experience was and who they observed.
2. Ask students to reflect on differences and/or similarities from what they saw in ICE to what they learned during their Doctoring course.
   - 1. Communication styles
     2. Patient involvement
     3. History taking or physical exams skills
3. Ask students what/if anything they saw during their ICE visits they plan to try and incorporate into their future care or if there were things they saw they want to make sure and not do in their future practice.
4. Expand the discussion by using any of the additional questions below.

**Guiding Questions**

Additional questions for discussion:

1. Were patients and families included in their own healthcare decisions? How did the inclusion or exclusion of patients and families in decision-making affect the healthcare they received?
2. Explore examples of high functioning teams:
   1. What made a team high functioning?
   2. What are some challenges to creating and maintaining high functioning teams (e.g., power dynamics/hierarchies, sexism/racism/etc.)?
   3. What could be done to improve team function?
3. Explore examples of interprofessional practice (IPP):
   1. What examples of interprofessional practice did you observe?
      1. Good or bad: example, inclusion of nursing in clinical decision making for patients
   2. What role was most surprising for you?
      1. e.g., pharmacist helps making treatment suggestions
   3. Did you observe any errors or near misses which were caught because of team based care or ones caught by either another member of the team or even a patient?
4. Were there any system-based issues that prevented a team from being high functioning, contributed to errors, or prevented patient and family centered care (PFCC)? e.g., nursing carried larger than normal load or worked longer than normal hours
