# Supplementary material for: The Interprofessional Clinical Experience: Introduction to Interprofessional Education Through Early Immersion in Health Care Teams
Source: MedEdPORTAL. 2017 Mar 30;13:10564. doi: 10.15766/mep_2374-8265.10564 (PMC6342292; doi:10.15766/mep_2374-8265.10564)
Supplement: Supplementary file 1 — A. ICE Instructor Packet.docx B. Prequiz.docx C. Clinical Introduction Session.docx D. Instructions for Video in Clinical Introduction.docx E. Video in Clinical Introduction Session.mp4 F. ICE Reading List.docx G. Reflection Assignment Instructions.docx H. Guide on How to Reflect.docx I. Experience and Reflection Notes.docx J. Small-Group Debriefing and Guiding Questions.docx K. Fall Semester Term Paper Instructions.docx L. Winter Semester Term Paper Instructions.docx M. Sample Preceptor Assessment Form.docx N. Sample Course Evaluation Form.docx [file mep-13-10564-s001.zip › K. Fall Semester Term Paper Instructions.docx]

**Appendix K: Fall Semester Term Paper** **Instructions**

**Faculty Instructions:** An end-of-term paper encourages deep introspection and synthesis of experiences with values. Share the following instructions with students, and ask them to submit a short paper about their first semester of ICE.

**Student Instructions:** The purpose of this 2-3-page paper is to encourage continued reflection upon your learning goals and clinical experience during the fall term. For this paper, consider your experience as a whole, and write about it. In your writing, address the following: how has your awareness of yourself and others as medical professionals changed over the course of the term? What trends have you noticed in communication, teamwork, or systems? What do you hope to learn as you enter the second term?
