# Supplementary material for: The Interprofessional Clinical Experience: Introduction to Interprofessional Education Through Early Immersion in Health Care Teams
Source: MedEdPORTAL. 2017 Mar 30;13:10564. doi: 10.15766/mep_2374-8265.10564 (PMC6342292; doi:10.15766/mep_2374-8265.10564)
Supplement: Supplementary file 1 — A. ICE Instructor Packet.docx B. Prequiz.docx C. Clinical Introduction Session.docx D. Instructions for Video in Clinical Introduction.docx E. Video in Clinical Introduction Session.mp4 F. ICE Reading List.docx G. Reflection Assignment Instructions.docx H. Guide on How to Reflect.docx I. Experience and Reflection Notes.docx J. Small-Group Debriefing and Guiding Questions.docx K. Fall Semester Term Paper Instructions.docx L. Winter Semester Term Paper Instructions.docx M. Sample Preceptor Assessment Form.docx N. Sample Course Evaluation Form.docx [file mep-13-10564-s001.zip › L. Winter Semester Term Paper Instructions.docx]

**Appendix L: Winter Semester Term Paper Instructions**

**Faculty Instructions:** An end-of-term paper encourages deep introspection and synthesis of experiences with values. Share the following instructions with students, and ask them to submit a short paper about their first semester of ICE.

**Student Instructions:** Similar to the fall end-of-term reflection paper, consider your experience as a whole and write about it. What was your overall experience in both clinical settings? What have you learned? How will your practice change as a result of this experience? How will you continue learning?
