# Supplementary material for: The Interprofessional Clinical Experience: Introduction to Interprofessional Education Through Early Immersion in Health Care Teams
Source: MedEdPORTAL. 2017 Mar 30;13:10564. doi: 10.15766/mep_2374-8265.10564 (PMC6342292; doi:10.15766/mep_2374-8265.10564)
Supplement: Supplementary file 1 — A. ICE Instructor Packet.docx B. Prequiz.docx C. Clinical Introduction Session.docx D. Instructions for Video in Clinical Introduction.docx E. Video in Clinical Introduction Session.mp4 F. ICE Reading List.docx G. Reflection Assignment Instructions.docx H. Guide on How to Reflect.docx I. Experience and Reflection Notes.docx J. Small-Group Debriefing and Guiding Questions.docx K. Fall Semester Term Paper Instructions.docx L. Winter Semester Term Paper Instructions.docx M. Sample Preceptor Assessment Form.docx N. Sample Course Evaluation Form.docx [file mep-13-10564-s001.zip › M. Sample Preceptor Assessment Form.docx]

**Appendix M: Sample Preceptor Assessment Form**

**Faculty Instructions:** After each ICE experience, students should seek feedback from the professional they observed. This feedback will evaluate them on their professionalism, communication skills, and teamwork.

**Student Instructions:** After your ICE experience, share this form with the professional you observed.

**Preceptor Instructions:** This form asks you to evaluate the first-year medical student shadowing you as part of the Interprofessional Clinical Experience (ICE). Your assessment will provide the student with feedback for improvement, and it may be used in assigning the student a Pass/Fail grade. Your assessment is not anonymous. Students can view your assessment once you complete it.

**Communication**

Please indicate the student’s competency in the area of communication.

|  | Area for Development | Appropriate for level | Notable Strength | Unable to Assess/Insufficient Data |
| --- | --- | --- | --- | --- |
| Communication with patients, families, public; socio-cultural awareness |  |  |  |  |
| Interprofessional communication |  |  |  |  |
| Sensitivity, honesty, compassion in communication |  |  |  |  |
| Understands own and others’ perspectives; how these influence communication |  |  |  |  |

Comment on the strengths / areas of improvement for students in this area.

|  |
| --- |

**Professionalism**

Please indicate the student’s competency in the area of professionalism.

|  | Area for Development | Appropriate for level | Notable Strength | Unable to Assess/Insufficient Data |
| --- | --- | --- | --- | --- |
| Demonstrates accountability to patients, society, and the profession. |  |  |  |  |
| Demonstrates compassion, integrity, respect, responsiveness to diverse populations. |  |  |  |  |

Comment on the strengths / areas of improvement for students in this area.

|  |
| --- |

**Leadership, Teamwork, and Interprofessionalism**

Please indicate the student’s competency in the areas of leadership, teamwork, and interprofessionalism.

|  | Area for Development | Appropriate for level | Notable Strength | Unable to Assess/Insufficient Data |
| --- | --- | --- | --- | --- |
| Understand one’s own and others’ roles to coordinate team-based care, providing care that is safe, efficient, effective, equitable |  |  |  |  |

Comment on the strengths / areas of improvement for students in this area.

|  |
| --- |

**Overall Assessment**

Comment on the overall strengths of this student.

|  |
| --- |

Comment on the overall areas for development.

|  |
| --- |

**Length of Contact with the Student** (Select one)

1 half day

2 half days

3 half days

4 half days
