# Supplementary material for: The Interprofessional Clinical Experience: Introduction to Interprofessional Education Through Early Immersion in Health Care Teams
Source: MedEdPORTAL. 2017 Mar 30;13:10564. doi: 10.15766/mep_2374-8265.10564 (PMC6342292; doi:10.15766/mep_2374-8265.10564)
Supplement: Supplementary file 1 — A. ICE Instructor Packet.docx B. Prequiz.docx C. Clinical Introduction Session.docx D. Instructions for Video in Clinical Introduction.docx E. Video in Clinical Introduction Session.mp4 F. ICE Reading List.docx G. Reflection Assignment Instructions.docx H. Guide on How to Reflect.docx I. Experience and Reflection Notes.docx J. Small-Group Debriefing and Guiding Questions.docx K. Fall Semester Term Paper Instructions.docx L. Winter Semester Term Paper Instructions.docx M. Sample Preceptor Assessment Form.docx N. Sample Course Evaluation Form.docx [file mep-13-10564-s001.zip › N. Sample Course Evaluation Form.docx]

**Appendix N: Sample Course Evaluation Form**

**Faculty Instructions:** The following sample course evaluation form can be used to evaluate ICE at various points of the academic year. The first set of questions can be distributed immediately following the Clinical Introduction session.

**Student Instructions:** Please indicate your level of agreement with the following statements about the Clinical Introduction Session:

|  | Strongly Disagree | Disagree | Neither Agree nor Disagree | Agree | Strongly Agree |
| --- | --- | --- | --- | --- | --- |
| Activities in the Clinical Introduction Session increased my understanding of the education and training required for the other health professions. |  |  |  |  |  |
| Activities in the Clinical Introduction Session increased my understanding of the roles other health professionals play in patient care. |  |  |  |  |  |

**Please provide any further comments you have about the Clinical Introduction Session:**

Please indicate your level of agreement with the following statements about your ICE experiences:

|  | Strongly Disagree | Disagree | Neither Agree nor Disagree | Agree | Strongly Agree |
| --- | --- | --- | --- | --- | --- |
| This course contributed to my understanding of healthcare team professions and roles. |  |  |  |  |  |
| This course improved my ability to communicate with healthcare professionals. |  |  |  |  |  |
| This course improved my understanding of healthcare systems. |  |  |  |  |  |
| This course improved my ability to work on an interprofessional team. |  |  |  |  |  |

**Please provide any further comments you have about the course:**
